# Maxilla reconstruction with 100% BioOss: A clinical and tomographic follow-up study

**DOI:** 10.4317/jced.61372

**Published:** 2024-09-01

**Authors:** Thiago-Revillion Dinato, José-Cicero Dinato, Fábio-Sá Carneiro Sczepanik, Márcio-Lima Grossi

**Affiliations:** 1 Clínica Dinato de Odontologia; 2 Professor, Post-Graduate Program in Dentistry School of Health and Life Sciences - Pontifical Catholic University of Rio Grande do Sul

## Abstract

**Background:**

Tooth loss and use of a complete denture is still a reality and results in bone loss. Adequate reconstruction of an extremely atrophic edentulous maxilla is a challenge, and different treatment methods have been described for its resolution.

**Material and Methods:**

Patients seeking implant placement in edentulous upper jaw with atrophic maxilla were selected in a private clinic in Porto Alegre, Brazil. The bone graft was performed with bilateral sinus lift and horizontal bone graft in anterior region with 0,25-1mm particles of Bio-Oss (Geistlich) covered with a collagen membrane (Bio-Gide, Geistlich). CBCTs were evaluated to verify the need for bone graft, and 6-8 months after bone graft follow-up, to plan implant placement and assess horizontal bone gain.

**Results:**

124 implants were placed in 19 patients, 76 of those in the sinus region. The survival rate was 95.2%, with six implants lost over a mean implants follow-up time of 47.68 months. The horizontal bone gain ranged from 0.00 to 6.86 mm, a mean gain of 2.85mm. An average of 5.5g of Bio-Oss was used per patient, and in 73.7 % of the cases, a flapless surgery was possible for implant placement, and in 92 implants an immediate loading was possible. Final rehabilitation was accomplished with fixed prosthodontics in 16 patients with a mean follow-up of 38.4 months.

**Conclusions:**

Within the limitations of this study, it is possible to affirm that bone graft with 100% Bio-Oss in atrophic maxilla is a reliable treatment and allow rehabilitation with implants with a high survival rate and the higher the initial bone height, the greater the gain in bone width.

** Key words:**Bone Regeneration, Dental Implants, Prosthodontics, Dentistry.

## Introduction

Dental rehabilitation of partially or totally edentulous patients with oral implants is a valid method for restoring oral aesthetics and function with predicTable results (1). A minimum amount of bone width and height is essential for the successful placement of implants (1,2). Unfavorable local conditions, due to atrophy, trauma and periodontal disease, may provide insufficient bone volume or an unfavorable interarch relationship, which does not allow a correct and prosthodontically guided positioning of dental implants (2). Thus, there are multiple etiologies for a patient to have a narrow bone in the anterior maxilla, and a successful implant therapy is dependent upon an adequate volume of bone at the site of implant placement (3).

Multiple restorative methods are available to restore the missing teeth in the anterior maxilla, including: a) implant-borne fixed restorations with or without prosthetic gingiva, b) fixed partial dentures supported by teeth, and c) removable options. To provide the satisfactory environment for an esthetic implant restoration, reconstruction of the alveolar ridge width needs to be accomplished in order to allow implant placement and to provide an ideal ridge contour for an esthetic appearance. The goal is to provide a reliable, minimally invasive, and long-term predicTable method to widen the narrow ridge in order to support dental implants and esthetic restorations (3).

Many techniques have been developed to reconstruct deficient alveolar jaws for the placement of dental implants performed either in combination or in second stage surgery after a period of healing, since adequate reconstruction of an extremely atrophic edentulous maxilla has always been a challenge (4). The lack of bone volume in combination with aging results in a change in facial morphology, which is often treated by a sinus lift combined with onlay bone grafting which, and for many surgeons is considered a reliable and predictable technique.

The development of the guided bone regeneration (GBR) technique started in the late 1980s, with a series of experimental studies trying to reduce morbidity to patients. The GBR is based on the concept of using either a resorbable or a non-resorbable barrier membrane in order to stabilize the blood clot and to create a space in which cells originating from bone tissue can grow without the interference of the faster proliferating soft tissue cells (4,5). The GBR also allows the ideal positioning of dental implants in atrophic ridges.

Scientific data regarding the amount of bone gain using biomaterials are scarce. Therefore, the aims of the study are: a) to describe a technique using 100% Bio-Oss (Geistlich) for maxilla reconstruction; b) to measure horizontal bone gain 6 to 8 months after bone graft in the anterior region of the maxilla; c) to relate the horizontal bone gain with the initial bone height; and d) to evaluate the survival rate and variables related to bone augmentation and/or implant survival rate.

The considered hypotheses were: a) an efficient treatment with DBBM and collagen membrane solely; b) high horizontal bone gain; and c) higher bone width gain in higher initial bone height; d) high survival rates for the implants

## Material and Methods

-Population, research design, inclusion/exclusion criteria, and blinding 

In this study, 18 to 85 year-old patients seeking implant placement in edentulous upper jaws with an atrophic maxilla in need of sinus lifting and ridge augmentation were selected in a private clinic in the city of Porto Alegre, Brazil. Both, the bone height and width of the crest were insufficient in dimension (i.e.: <4mm width; <7mm height) for conventional implant placement. Hence, a GBR procedure aimed at augmentation of the ridge was included in the treatment plan beyond the sinus lift. Patients with blood disorders, uncontrolled diabetes, smoking, history of previous surgery, and presence of any pathology in the sinus were excluded. After clinical and radiographic evaluation, the patients read and signed the information consent form regarding the surgical procedure, including its advantages and disadvantages. The implant specialist JCD, who performed all bone grafts, did not participate in the data analysis. The study protocol was reviewed and approved by the Research Ethics Committee of the Pontifical Catholic University of Rio Grande do Sul (CEP/PUCRS, No #1.892.269), State of Rio Grande do Sul, Brazil.

-Clinical Variables

The following data were gathered for each patient: a) age, b) sex, c) DBBM quantity per patient, d) implant features, e) timing of implant placement, f) flap elevation, and g) timing of prosthetic rehabilitation. The implant features included both its length (i.e.: 8, 10, 11.5, 13 mm and 16mm) and diameter (i.e.: 4.3 mm). The implants were placed either after a conventional mucoperiostal flap or a guided surgery flapless technique. All implants were installed using a precision guide for determining its position and depth (i.e., 1mm subcrestal).

-Ridge Augmentation Procedures

**Figure 1:**
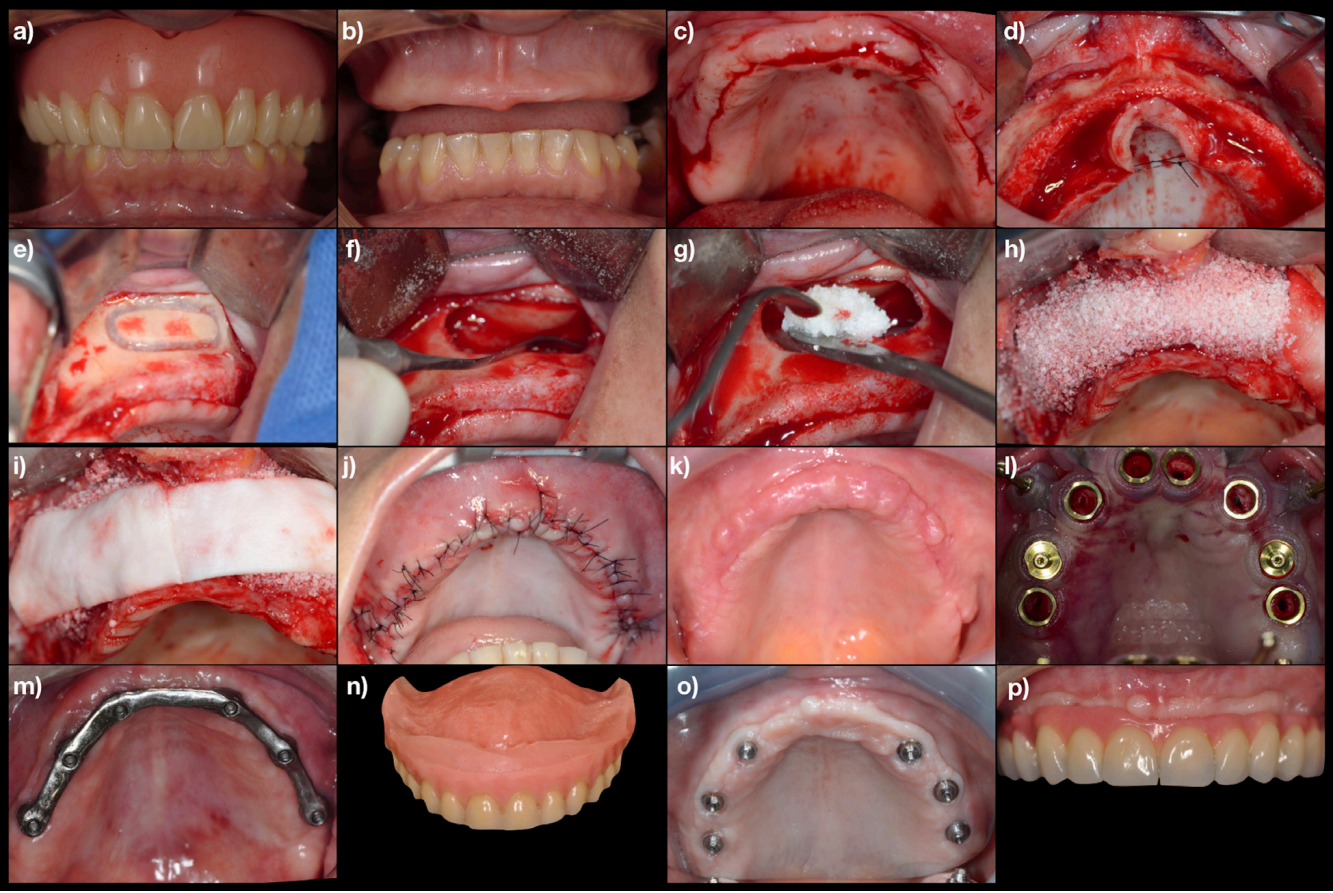
a) Intraoral aspect with prosthesis in position; b) Initial intraoral aspect; c) Line of incision towards the palatal aspect of the ridge in the maxilla with oblique-releasing incisions; d) Mucoperiosteal flap raised; e) Lateral window to the maxillary sinus; f) Deflection of Schneider’s membrane; g) Maxillary sinus filled with small particles of biomaterial; h) Horizontal increase in the anterior region with Bio-Oss (Geistlich); i) Resorbable collagen membrane covering the grafted region; j) Horizontal mattress sutures and single interrupted sutures; k) Intraoral aspect after 6 months; l) Guided surgery for implants placement; m) Milled bar connecting implants for immediate loading with temporary overdenture; n) Full denture with resilient material; o) Intraoral aspect 6 months after implant installation; p) Fixed final prosthesis over the implants.

The need for bone graft (i.e., native bone width ≤4mm) was determined after a clinical evaluation with cone beam computed tomography (CBCT). Before the surgical procedure for sinus lifting and ridge augmentation, the patients were given 2g of amoxicillin (Fig. 1a). Following a mouth rinse with 0.12% of an aqueous solution of chlorhexidine, the area intended for surgery was carefully anesthetized using local anesthetics (Fig. 1b). To raise a mucoperiosteal flap, a paracrestal technique was used placing the line of incision towards the palatal aspect of the ridge in the maxilla. Oblique-releasing incisions were used to allow for a wide flap basis and sufficient access to the defective ridge area (Fig. 1c). The flaps were carefully raised using tissue elevators. The bone ridge was examined and any soft tissues remaining on the crest were meticulously removed with a surgical curette (Fig. 1d).

The lateral window was established in an oval shape using a #6 round diamond bur (Fig. 1e). The sinus membrane was deflected (Fig. 1f), and the space created was filled with small (0.25 – 1 mm) DBBM particles (Fig. 1g), which has been shown in the literature to have higher osteoconduction (6). If the membrane was perforated or torn, a collagen membrane was used to repair the damage. The aim was to increase the bone height to a sufficient size for an 8mm implant placement, or higher. The graft particles were positioned into the sinus cavity and in the defect area (Fig. 1h). The aim was to increase the ridge width to a size sufficient for standard implant placement (i.e., 4mm or more). The membrane-supporting material was partly stabilized by the morphology of the ridge, and partly by the covering membrane. A collagenous membrane was trimmed to cover the membrane-supporting material and to extend it 2 mm on the intact bone borders of the defect (Fig. 1i). Releasing incisions were made through the periosteum at the base of the flap in order to allow tension-free adaptation of the wound margins. Horizontal mattress sutures as well as single interrupted or continuous sutures were placed to achieve healing by primary intention (Fig. 1j). The patients received prescriptions for analgesic (500mg of acetaminophen a day), anti-inflammatory (200mg of nimesulide a day, for 5 days), and antibiotic (1,500mg of amoxicillin a day, for 7 days) therapies. The patients were instructed to rinse with a 0.12% solution of chlorhexidine twice a day for 2 weeks, starting on the day after the surgery. Temporary dentures were not used for at least two weeks. Ten days following augmentation surgery, the interrupted sutures were removed. Follow-up visits were scheduled every 4-6 weeks until re-entry surgery with clinical and radiographic evaluation.

Six to eight months following augmentation surgery, a clinical evaluation (Fig. 1k) with CBCT was performed to analyze bone availability; and implantation surgery was carried out. Patients were scheduled for implant placement (Fig. 1l). All implants were used with a Morse taper connection and were placed 1 mm subcrestally in the previously planned position (i.e., corresponding to the future crown center). All implants used in this study had a full sandblasted and acid-etched (NeoPoros) surface treatment. An immediate loading with a bar connecting the implants (Fig. 1m) and a provisional denture (Fig. 1n) over it was performed when 4 or more implants presented at least 32N, when not, patients underwent a second stage surgery for abutment placement and oral rehabilitation after 6 months (Fig. 1o).

-Follow-Up

After the final prosthodontic treatment (Fig. 1p), patients were included in a maintenance program with recall appointments every 6 months. Periapical X-rays were taken, and clinical evaluation examined mobility, pain and/or infection associated with the implants. Cases were considered successful in the absence of pain or mobility upon re-entry and at recall appointments.

-Measurement technique

Diagnosis and pre-implant planning involved clinical examination and CBCT, which were taken with the IS i-CAT (version 17- 19, Imaging Sciences International). The following parameters were established: a) 120 kV, b) 5 mA, c) axial slice distance 0.300 mm3, and d) 23-cm field of view.

**Figure 2:**
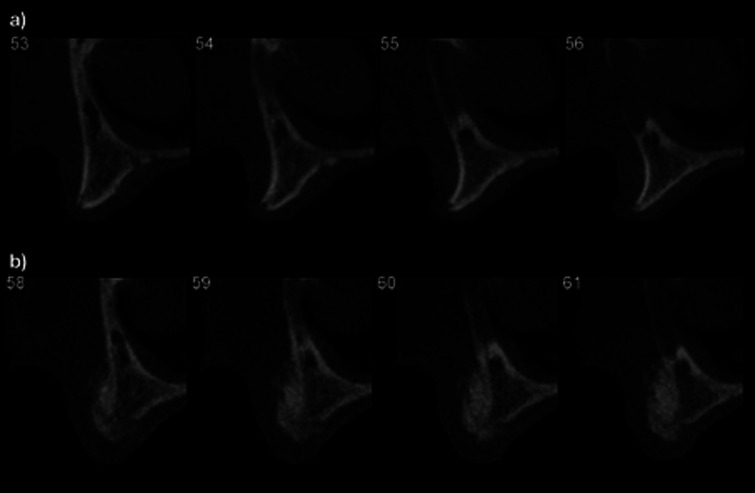
Tomographic images to be superposed for subsequent measurements. a) CBCT before bone graft, b) CBCT before implant placement.

**Figure 3:**
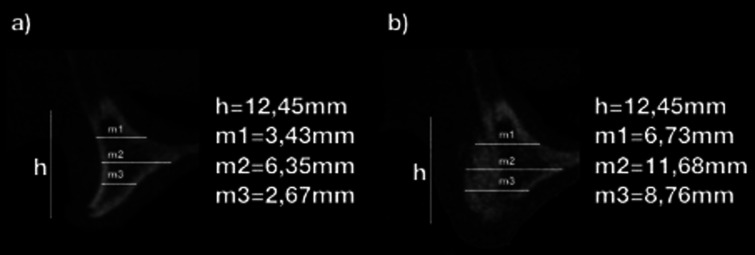
Measures in the CBCT before bone graft (a) and before implant placement (b).

**Figure 4:**
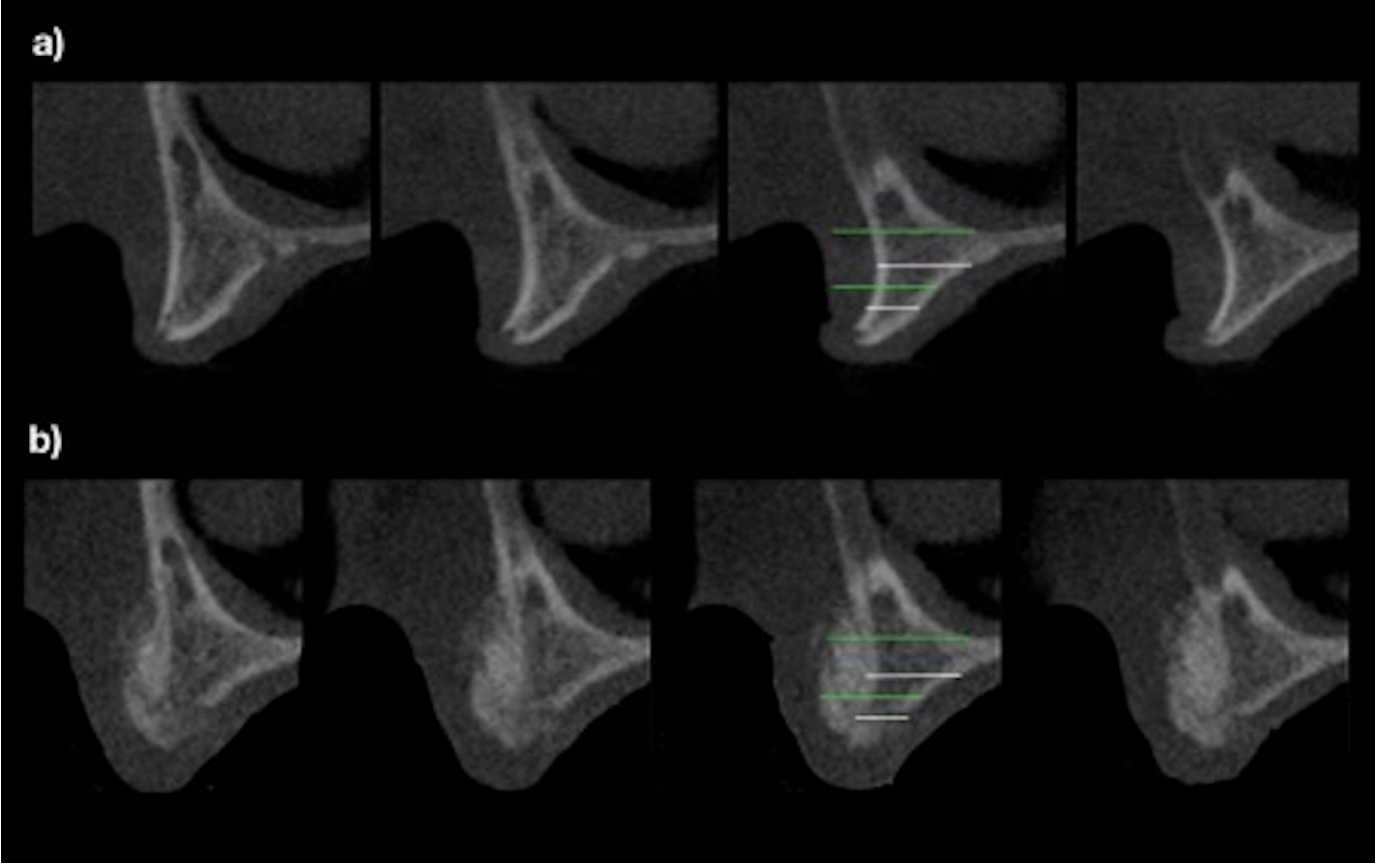
White line showing the measurement in the CBCT before bone graft (a) and green line showing the measurement in the CBCT before implant placement (b) superimposed on both tomographies to facilitate visualization of the bone gain.

Horizontal gain in bone width was calculated by comparing the CBCT taken before and 6 to 8 months after surgical intervention (Fig. 2); the evaluation was made by measuring bone width at 12 predetermined sites (i.e.: 3 on the right canine, 3 on the right central incisor, 3 on the left central incisor and 3 on the left canine area) that were the same in the 2 tomography taken. A digital superimposition was used to confirm the evaluation of the same sites (Figs. 3,4). All evaluations were repeated twice in 2 different days, and 12 measurements per patient were considered (i.e.: right canine, right central incisor, left central incisor and left canine area).

-Statistical analysis

SPSS® version 17 was used for the statistical analysis. The Kolmogorov-Smirnov normality test and the Levene’s homogeneity of variance test were used. Considering that all results had a parametric distribution, the Student’s paired and independent t tests, and repeated-measures ANOVA were used.

## Results

In 19 selected patients (6 males and 13 females; age range: 48 to 77 years; mean age: 61.4 years), 124 implants installed; 76 implants were placed in the grafted sinus and 48 in the anterior maxilla.  Table 1 shows description of surgical characteristics with an implant survival rate of 95.2% with six implants lost, which occurred 7 months after being installed, on average. Two of the lost implants were placed in the sinus. The implants lost were 8mm long (n=3), 10mm long (n=1), 11.5mm long (n=1) and 13mm long (n=1). Four out of the six implants were replaced at the moment of its removal, while the other two were not ( Table 2). All lost implants, except one, were installed during a flapless surgery and received immediate loading.

Ninety-two implants were inserted during a flapless surgery (74.2%), while the other 25.8% were inserted after raising a flap. Thus, in 14 patients the implants were installed with a guided surgery (73.7%) and the other 26.3%, in a conventional open flap way.

It was used an average of 5.5 ± 1.4g of DBBM per case, varying from 3g to 8g. Only one sinus out of 38 had a membrane perforation (2.6%), which was covered by a collagen membrane, and it was possible to perform the sinus graft.

The mean time for implant placement after the bone graft was 7.1 ± 2.1 months, while the mean time for rehabilitation after implant placement was 9.4 ± 3.9 months. The mean follow-up time was 47.7 months (3.9 years) for implants and 38.4 months (3.2 years) for the oral rehabilitation.

The oral rehabilitation was performed over multiple abutments with a fixed prosthesis over the implants in 16 patients (84.2%) and over a bar connecting the multiple abutments with an overdenture in the other 3 patients (15.8%).

All implants had 4.3 mm diameter and 71 were 13 mm long (57%), 60 of those were placed in the grafted sinus. In 14 patients an immediate loading with a bar connecting the implants and a provisional denture over it was possible (73.7%), while the other 5 patients underwent a second stage surgery for abutment placement and oral rehabilitation after 6 months. This type of immediate loading was performed when 4 or more implants presented at least 32N.

 Table 3 shows a significant statistical difference (*p* value < 0.001) when comparing the bone width before and after bone graft with DBBM.  Table 4 shows the comparison of gain in bone width after bone graft between different variables, the quantity of Bio-Oss used in the graft and the age of patients did not showed significant statistical differences. Also, bone grafts that posteriorly allowed flapless surgery, immediate loading, or that resulted in implant loss, did not show any statistical difference. On the other hand, patient’s gender resulted in significant difference (*p* value < 0.001), with a greater bone width gain in men (3.71 ± 1.62mm) as compared to women (2.38 ± 1.40mm).

Regarding the bone height prior to bone graft, it is possible to observe that the higher the bone, the more likely is to gain in width with the bone graft. This is shown in  Table 5 where a significant statistical difference is observed between different initial bone heights. The mean bone width gain was higher when the initial bone height was higher than 8 (3.51 ± 1.36 mm), 10 (3.71 ± 1.39 mm) or 12mm (4.10 ± 1.36 mm) compared to initial bone heights lower than 8 (1.46 ± 1.15 mm), 6 (1.17 ± 0.98 mm) or 4mm (1.02 ± 0.96 mm), respectively. Concerning sinus floor elevation, all of the sites had less than 4mm in height and the implants placed were 16mm long (5), 13mm long (60), 11,5mm long (9) and 10mm long (2).

## Discussion

-Graft Material

The present study performed all bone grafts with 100% DBBM, which is a proven evidence-based method of treatment according to Sanz *et al*. (7) Alluden *et al*. (8), in a systematic review comparing Bio-Oss alone and Bio-Oss mixed with particulate autogenous bone graft in lateral ridge augmentation, affirms that non comparative studies seems to indicate that both treatment facilitates formation of new bone, have postoperative dehiscence as a common complication, and have similar bone resorption and implant survival rates.

Some studies demonstrated that Bio-Oss is a non-resorbable or slowly resorbed bone substituten (9,10). However, this is not in accordance with Mordenfeld *et al*., who showed a two-dimensional width reduction with different mixtures of Bio-Oss and particulate autogenous bone varying between 27% and 47% after 7.5 months, which might be due to displacement or pressure from the soft tissue or the removable denture during mastication (11).

Onlay grafting with a cortical block has been evaluated over time, and the resorption of the graft cortical thickness has been reported to be as little as 1.2 mm to more than 50% of the graft thickness, which shows that volume loss occurs during this process, and it is difficult to predict (12-15).

Jemt and Lekholm (16) concluded that after 6 months, autogenous bone grafting can create sufficient bone volume for implant placement, but the individual variation in resorption pattern makes the grafting procedure unpredictable for the long-term prognosis.

When comparing the rate of graft resorption in autogenous iliac bone graft and guided bone regeneration in patients with atrophic maxilla in a retrospective study with 39 patients, Gultekin *et al*. showed that both materials can provide adequate volume for implant placement, but the autogenous bone graft results in greater bone resorption (17). These authors state that one of the reasons to have less bone resorption compared to other studies (14,15,18) might be a healing period of 3 months after bone graft as well as an additional 3 months healing for the implant osseointegration.

Allografts were also studied when Aslan *et al*. evaluate the clinical and histomorphometric features of demineralized freeze-dried cortical block allografts (DCBA) used for ridge augmentation. No membranes were used, and all cases were performed with a 2-stage approach (implant placement after 5 months of healing). Clinical analysis showed that the mean gain in horizontal bone was 1.65 ± 0.14 mm, and that the mean percentage of graft resorption was 5.39 ± 2.18% (19). In spite of the good results, allografts have the same problems of the autogenous grafts, since they resorb the same way.

-Implant Stability

Al-Khaldi *et al*. assessed the stability of dental implants placed in grafted versus nongrafted bone in the anterior maxilla using resonance frequency analysis (20). These authors found that implants placed in grafted bone compared favorably with those in nongrafted bone and showed excellent stability. This is in accordance with the present study, which showed high primary stability and led to an immediate loading in 14 cases. The reason to place a bar and a provisional overdenture instead of making a fixed prosthesis right after the surgery is to reduce the load on each implant, sharing it with the mucosa.

Hernández-Alfaro *et al*. also showed similar results after 14 edentulous patients were treated with bilateral sinus floor elevation, mandibular bone block grafts and biomaterials (21). In 81 of the 108 implants placed, it was possible to place them in immediate loading. The implant placement was performed 14 to 16 weeks after the bone graft surgery, with immediate loading in 10 patients.

The stability of implants placed in particulate bone, onlay block bone, interpositional bone, and nongrafted maxillary bone was also compared by Rasmuson *et al*. (22) during the early phase of osseointegration, by means of resonance frequency analysis and implant failure as endpoints. Implants placed in nongrafted and grafted maxillary bone using a two-stage protocol showed similar stability during the early phase of osseointegration.

-Survival Rate

The 95.16% implant survival rate presented in this study is lower than showed by studies assessing implants in sinus lift technique (23,24) and grafts with iliac crest or DBBM mixed with autogenous bone for atrophic maxilla (17), but it is in accordance Hellem *et al*. (25).

Jensen and Terheyden (26) concluded that a high level of evidence has shown that the survival rates of implants placed in augmented bone are comparable to the rates of implants placed in native bone.

The survival rates of implants placed in augmented sites with GBR are reported in many publications, several experimental studies (27,28). Studies evaluating clinical outcomes of lateral ridge augmentation with GBR procedures in staged implantation usually used autogenous bone as filler materials in combination with non-resorbable membranes (29,30). Limited data are available reporting on the application of bone substitutes in combination with resorbable membranes for ridge augmentation before implant installation. However, in 2008, Meijndart *et al*. (31) concluded that xenografts were equivalent to autogenous bone grafts when evaluating both implant survival and the peri-implant hard/soft tissue reactions.

-Implant timing

Clementini *et al*., in a systematic review, showed that, despite no studies presenting a control group and a standardized success criterion are found, the delayed positioning of implants should be considered more predictable than the immediate positioning (32). The study assessed maxillary and mandibular bone grafts with different types of augmentations, but their results were in agreement with the present study, where the implant surgery was performed in a second stage in all patients.

Aloy-Prósper *et al*. (33) also compared implant timing in a 3-year retrospective study with intraoral onlay block bone grafts. A total of 53 implants (23 delayed and 30 simultaneous) were included, and the cumulative implant success rate was 83.3% for simultaneous and 96.9% for delayed implants, which corroborates with the another study (32)

-Bone gain

Most studies (17,21,34) showed a very pronounced bone resorption during healing before implant placement, giving the reason why the measurements were made 6-8 months after the bone graft. Thus, the stability of the augmented site in this period is an important factor in the maintenance of graft sites in the following years (17).

To assess horizontal bone augmentation, Qiu and Yu evaluated onlay bone graft with DBBM block and autogenous bone in the anterior maxilla in a prospective study including 14 patients (34). The authors also used particulate DBBM and a double layer of collagen membrane; and they reported a width gain of 8.73mm, but with a resorption rate of 7.03%. The oro-facial bone width was measured using a calibrated caliper both at 1 mm below the highest point of the remaining crest before graft, and in the implant placement surgery. The present study reported a mean gain in bone width of 2.85 ± 1.44mm performing the bone graft only with DBBM particles and collagen membrane.

Hämmerle *et al*. reported a mean ridge width gain of 3.6mm after a bone graft with both particles or blocks of DBBM (Bio-Oss) and a collagen membrane (Bio-Gide), with only one failure (35). The augmented areas included one to multiple teeth and it was observed an integration of the DBBM particles into the newly formed bone. The present study performed a flapless surgery in most of the cases in the second stage, but that’s what was seen in the open flap implant placement surgeries.

Another study assessed gain in bone volume after patients underwent bone graft with autogenous bone block, DBBM particles and collagenous membrane. The average percentage volumetric increase between the preoperative condition and the situation at reentry was 71.99% (21). Despite not evaluating the bone gain in volume, it is possible to affirm that a horizontal bone gain of 63.62% was achieved in our study.

When comparing block grafts harvested from iliac crest (IC) or mandibular ramus (MR), both combined with DBBM particles and collagen membrane, for horizontal bone augmentation, Monje *et al*. shows that IC leads to a greater ridge width gain than MR (4.93mm vs 3.23mm) (36). All cases were performed in severe maxillary anterior ridge defects and the results are in accordance with other studies (34,35).

## Conclusions

Within the limitations of this study it is possible to affirm that: 1) the bone graft with 100% DBBM in atrophic maxilla is a reliable treatment; 2) the 95.2% survival rate found encourages placement of implants in reconstructed maxillae; 3) a predictable horizontal bone gain is achievable; 4) the higher the initial bone height, the higher the possibility in bone width gain; 5) flapless surgery for implant placement is commonly an option; 6) immediate loading is achievable in most of the cases; and 7) the replacement of lost implants is possible. More studies are necessary to confirm this data with randomized trials and long-term analysis.

## Figures and Tables

**Table 1:** Description of surgical characteristics (n = 124 implants, n = 19 patients, n = 38 sinuses).

Bio-Oss quantity (g) Mean (±SD)	5.5 (1.4)
Time of implant placement after bone graft (months) Mean (±SD)	7.1 (2.1)
Time of prosthesis placement after implant placement (months) Mean (±SD)	9.4 (3.9)
Maxillary sinus membrane perfuration (%) Yes No	(n=38) 2.6 97.4
Implant placement surgery (%) Flapless Open flap	(n=124) 73.7 26.3
Loss of implant(s) after placement (%) Yes No	(n=124) 4.8 95.2
Follow-up time after implant placement (months) Mean (±SD)	47.7 (20.3)
Follow-up time after prosthesis placement (months) Mean (±SD)	38.4 (22.2)
Immediate implant loading after surgery (%) Yes No	(n=124) 73.7 26.3
Horizontal bone gain (mm) Mean (±SD)	2.85 (1.44)

**Table 2:** Description of implants placed (n = 124).

Number of implants per patient Mean (±SD)	6.5 (0.9)
Size of implants placed (count) 4.3 x 16.0 4.3 x 13.0 4.3 x 11.5 4.3 x 10.0 4.3 x 8.0	(n=124) 5 71 17 16 15
Size of implants lost (count) 4.3 x 16.0 4.3 x 13.0 4.3 x 11.5 4.3 x 10.0 4.3 x 8.0	(n = 6) 0 1 1 1 3
Replacement(s) of lost implants (%) Yes No	(n = 6) 66.7 33.3
Time until loss of implant(s) (months) Mean (±SD)	(n = 6) 7.2 (4.8)

**Table 3:** Comparison of bone width (mm) before and after bone graft between different variables.

Independent variables:	Initial bone width Mean ±SD	Final bone width Mean ±SD	p value
General mean bone width	(n=228) 4.48 ±1.09	(n=228) 7.33 ± 2.00	<0.001*
Initial bone height greater than 12 mm	(n=60) 4.76 ± 2.06	(n=60) 8.86 ± 2.96	<0.001*
Initial bone height greater than 10 mm	(n=102) 4.54 ± 1.91	(n=102) 8.25 ± 2.70	<0.001*
Initial bone height greater than 8 mm	(n=162) 4.58 ± 1.89	(n=162) 8.09 ± 2.65	<0.001*
Initial bone height less than 8 mm	(n=66) 4.27 ± 2.00	(n=66) 5.74 ± 2.69	<0.001*
Initial bone height less than 6 mm	(n=39) 3.96 ± 1.98	(n=39) 5.13 ± 2.67	<0.001*
Initial bone height less than 4 mm	(n=27) 3.48 ± 1.54	(n=27) 4.50 ± 2.27	<0.001*
≤5 g BioOss	(n=108) 4.49 ± 1.99	(n=108) 7.21 ± 2.81	<0.001*
> 5 g BioOss	(n=120) 4.47 ± 1.87	(n=120) 7.44 ± 2.93	<0.001*
Women	(n=156) 4.21 ± 1.79	(n=156) 6.59 ± 2.54	<0.001*
Men	(n=72) 4.98 ± 2.08	(n=72) 8.69 ± 2.96	<0.001*
≤60 y.o.	(n=96) 4.67 ± 1.85	(n=96) 7.49 ± 2.31	<0.001*
>60 y.o.	(n=132) 4.37 ± 1.99	(n=132) 7.32 ± 3.28	<0.001*
Open flap surgery	(n=60) 4.67 ± 2.03	(n=60) 7.63 ± 2.59	<0.001*
Flapless surgery	(n=168) 4.40 ± 1.88	(n=168) 7.21 ± 2.98	<0.001*
Immediate implant loading	(n=168) 4.50 ± 1.96	(n=168) 7.34 ± 3.03	<0.001*
Delayed implant loading	(n=60) 4.41 ± 1.82	(n=60) 7.30 ± 2.29	<0.001*
Patients with implant loss	(n=60) 4.27 ± 1.88	(n=60) 7.14 ± 2.97	<0.001*
Patients without implant loss	(n=168) 4.57 ± 1.94	(n=168) 7.41 ± 2.84	<0.001*

**Table 4:** Comparison of gain in bone width (mm) after bone graft between different variables.

Independent variables	Mean bone gain	Standard deviation	p value
≤ 5 g BioOss (n=108)	2.72	1.53	NS^*^
> 5 g BioOss (n=120)	2.96	1.68	
Women (n=156)	2.38	1.40	<0.001*
Men (n=72)	3.71	1.62	
≤ 60 y.o. (n=96)	2.82	1.31	NS*
> 60 y.o.(n=132)	2.94	1.87	
Open flap surgery (n=60)	2.95	1.57	NS*
Flapless surgery (n=168)	2.80	1.63	
Immediate implant loading (n=168)	2.83	1.66	NS*
Delayed implant loading (n=60)	2.89	1.46	
Patients with implant loss (n=60)	2.87	1.68	NS*
Patients without implant loss (n=168)	2.84	1.59	

**Table 5:** Comparison of bone width gain (mm) among different bone heights prior to bone graft.

Independent variables:	Mean bone gain	Standard Deviation	p value
Initial bone height >12 mm (n=60)	4.10 ^a^	1.36	<0.001*
Initial bone height >10 mm (n=102)	3.71 ^a^	1.39	
Initial bone height >8 mm (n=162)	3.51 ^a^	1.36	
Initial bone height <8 mm (n=66)	1.46	1.15 ^b^	
Initial bone height <6 mm (n=39)	1.17	0.98 ^b^	
Initial bone height <4 mm (n=27)	1.02	0.96 ^b^	

## Data Availability

The datasets used and/or analyzed during the current study are available from the corresponding author.
